# Supplementary material for: Rifabutin-Loaded Nanostructured Lipid Carriers as a Tool in Oral Anti-Mycobacterial Treatment of Crohn’s Disease
Source: Nanomaterials (Basel). 2020 Oct 27;10(11):2138. doi: 10.3390/nano10112138 (PMC7692220; doi:10.3390/nano10112138)
Supplement: Supplementary file 1 [file nanomaterials-10-02138-s001.pdf]

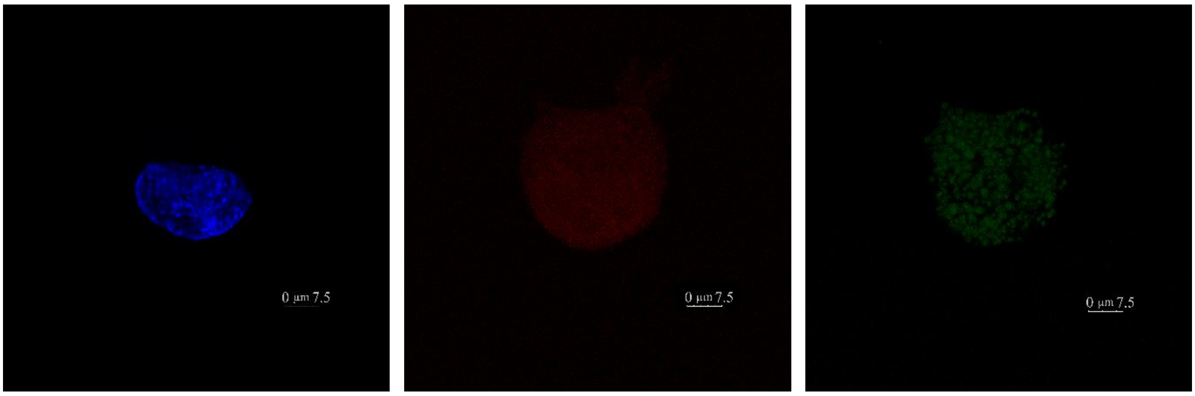

(**a**) (**b**) (**c**)

Figure S1. Confocal imaging of blank NLC macrophages uptake. Red, blue, and green colours represent cell cytoplasm (Alexa Fluor™ 647 phalloidin), cell nuclei (DAPI) and NLC formulations (Coumarin 6), respectively: (a) blue channel (b) red channel and (c) green channel.

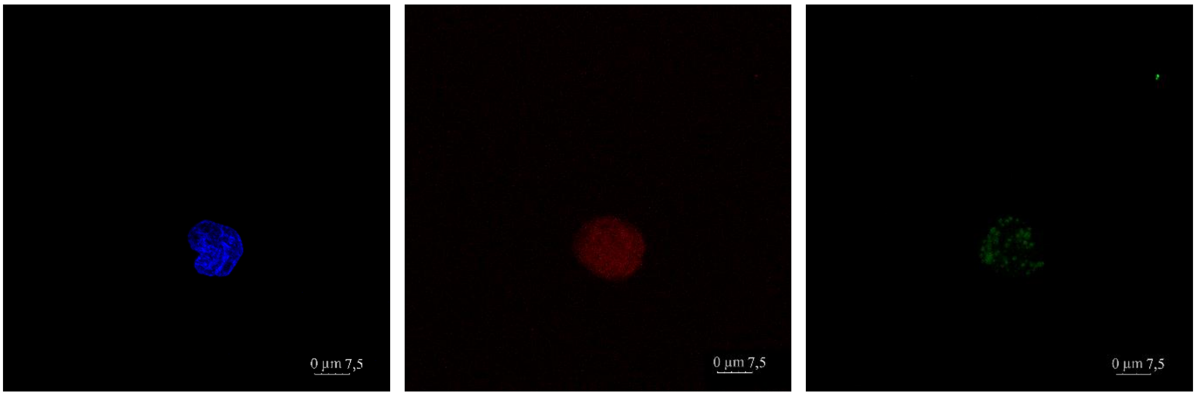

(a) (b) (c)

Figure S2. Confocal imaging of RFB-loaded NLC macrophages uptake. Red, blue, and green colours represent cell cytoplasm (Alexa Fluor™ 647 phalloidin), cell nuclei (DAPI) and NLC formulations (Coumarin 6), respectively: (a) blue channel (b) red channel and (c) green channel.

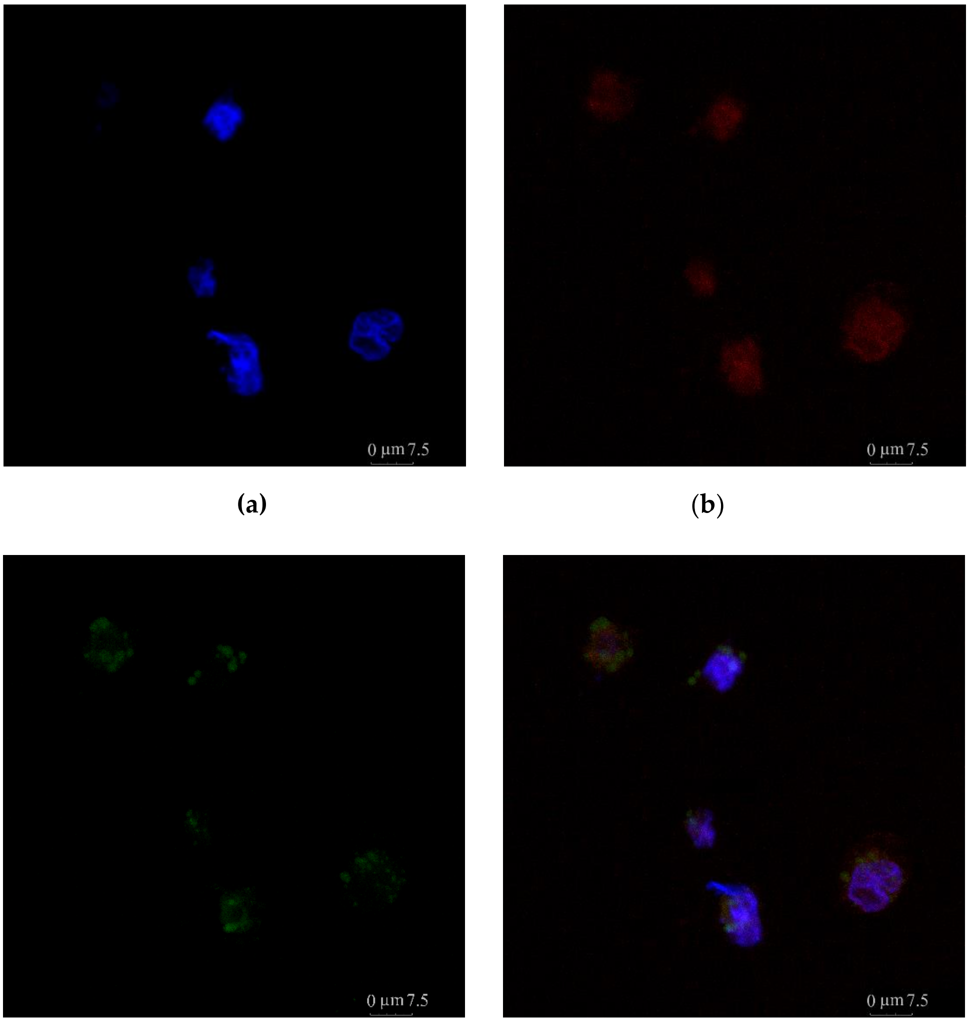

(**a**) (**b**)

(**c**) (**d**)

Figure S3. Confocal imaging of blank NLC uptake by a group of macrophages. Red, blue, and green colours represent cell cytoplasm (Alexa Fluor™ 647 phalloidin), cell nuclei (DAPI) and NLC formulations (Coumarin 6), respectively: (a) blue channel (b) red channel, (c) green channel and (d) overlay of the three channels.
